# Efficacy and Safety of Radiofrequency Ablation vs. Endoscopic Surveillance for Barrett’s Esophagus With Low-Grade Dysplasia: Meta-Analysis of Randomized Controlled Trials

**DOI:** 10.3389/fonc.2022.801940

**Published:** 2022-02-28

**Authors:** Yizi Wang, Bin Ma, Shize Yang, Wenya Li, Peiwen Li

**Affiliations:** ^1^Department of Obstetrics and Gynecology, Shengjing Hospital of China Medical University, Shenyang, China; ^2^Department of Colorectal Surgery, Cancer Hospital of China Medical University, Liaoning Cancer Hospital and Institute, Shenyang, China; ^3^Department of Thoracic Surgery, The First Hospital of China Medical University, Shenyang, China

**Keywords:** Barrett’s esophagus, radiofrequency ablation, endoscopic surveillance, high-grade dysplasia (HGD), esophageal adenocarcinoma (EAC), low-grade dysplasia (LGD)

## Abstract

**Background and Aims:**

Barrett’s esophagus with low-grade dysplasia (BE-LGD) carries a risk of progression to Barrett’s esophagus with high-grade dysplasia (BE-HGD) and esophageal adenocarcinoma (EAC). Radiofrequency ablation (RFA) appears to be a safe and efficacious method to eradicate Barrett’s esophagus. However, a confirmed consensus regarding treatment of BE-LGD with RFA vs. endoscopic surveillance is lacking. Therefore, this study aimed to elucidate the efficacy and safety for RFA vs. endoscopic surveillance in decreasing the risk of BE-LGD progression to BE-HGD or EAC.

**Methods:**

Relevant studies published before May 1, 2021 were identified by searching relevant medical databases. The primary outcome was the rate of progression BE-LGD to HGD and/or EAC after treatment with RFA and endoscopic surveillance. The secondary outcome was the rate of complete eradication of dysplasia (CE-D) and complete eradication of intestinal metaplasia (CE-IM) after treatment with RFA and endoscopic surveillance. Adverse events were also extracted and evaluated.

**Results:**

Three randomized controlled trials were eligible for analysis. The pooled estimate of rate of neoplastic progression of BE-LGD to HGD or EAC was much lower in the RFA group than the endoscopic surveillance group (RR, 0.25; 95% CI, 0.07–0.93; P = 0.04), with moderate heterogeneity (I^2^ = 55%). Subgroup analysis based on progression grade was performed. The pooled rate of progression of BE-LGD to HGD was much lower in the RFA group than the endoscopic surveillance group (RR, 0.25; 95% CI, 0.07–0.71; P = 0.01), with low heterogeneity (I^2^ = 15%). Although the pooled risk of progression of BE-LGD to EAC was slightly lower in the RFA group than the endoscopic surveillance group (RR, 0.56; 95% CI, 0.05–6.76), the result was not statistically significant (P = 0.65). RFA also was associated a higher rate of CE-D and CE-IM both at the end of endoscopic treatment and during follow-up. However, the rate of adverse events was slightly higher after RFA treatment.

**Conclusion:**

RFA decreases the risk of BE-LGD progression to BE-HGD. However, given the uncertain course of LGD and the potential for esophageal stricture after RFA, treatment options should be fully considered and weighed.

**Systematic Review Registration:**

https://www.crd.york.ac.uk/prospero/display_record.php?ID=CRD42021266128, identifier PROSPERO (CRD42021266128).

## Background

Barrett’s esophagus (BE) results in a significant histological change in which the normal squamous epithelium is replaced by columnar epithelium, an outcome known as intestinal metaplasia (1). This metaplastic change is caused by gastroesophageal reflux disease (2). The prevalence of BE in Europe and the USA has been estimated to be 1.6%, and 1.7–5.6%, respectively (2). The neoplastic progression of BE ranges from nondysplastic intestinal metaplasia to low-grade dysplasia (LGD), high-grade dysplasia (HGD) and eventually esophageal adenocarcinoma (EAC).

According to a previous study, the neoplastic progression of BE results in nondysplastic intestinal metaplasia or transient LGD in most cases (3), it can also lead to HGD and EAC. Hence, endoscopic surveillance is advisable for most patients. For HGD and EAC, the consensus treatment is endoscopic resection of visible lesions and radiofrequency ablation (RFA) of residual BE (4, 5). However, for BE with LGD (BE-LGD), some uncertainties exist regarding diagnosis and the natural disease course: some cases may progress to HGD or EAC, whereas others may remain stable, degenerate or even result in nondysplastic intestinal metaplasia (6). Therefore, whether BE-LGD should be treated with RFA or just endoscopic surveillance remains an open question.

Few studies have compared the risk of progression to HGD or EAC after treatment with RFA vs. endoscopic surveillance in patients diagnosed with BE-LGD (7, 8). However, retrospective results could not provide a convincing conclusion because of the heterogeneous study characteristics.

Recently, several RCTs have evaluated the risk of BE-LGD progression to the next neoplastic stage between endoscopic RFA and surveillance. However, the number of cases in these studies has been limited, and the results have been somewhat inconsistent.

The aim of this meta-analysis was to evaluate the efficacy and safety of RFA compared with endoscopic surveillance in reducing the risk of progression of BE-LGD to BE-HGD or EAC.

## Methods

### Study Searching and Search Strategy

Three databases (PubMed, the Cochrane Central Register of Controlled Trials and Web of Science) were searched for eligible publications according to the Preferred Reporting items for Systematic Review and Meta-Analysis (PRISMA) guidelines (9). The most recent search was performed on May 1, 2021. The full search strategies for the three databases are presented in **Supplementary Table 1**. A PRISMA checklist is provided in **Supplementary Table 2**. The time period was not limited. Additional studies were identified by searching the remaining articles after exclusion of those unrelated to our questions of interest. The protocol of this meta-analysis was registered at PROSPERO (CRD42021266128).

### Inclusion and Exclusion Criteria

Two investigators (YZ.W. and B.M.) independently screened all relevant studies and reviewed the full text of the included studies. Any disagreements were discussed with a third reviewer (PW.L.) and resolved by consensus.

The inclusion criteria were as follows: (1) randomized controlled trials (RCTs); (2) studies presenting clinical data on patients with a confirmed diagnosis of BE-LGD treated with RFA or endoscopic surveillance, e.g., the rate of progression to HGD and/or EAC, rate of complete eradication of dysplasia (CE-D) or rate of complete eradication of intestinal metaplasia (CE-IM).

The exclusion criteria were as follows: (1) fewer than ten cases in the study; (2) treatment (RFA or surveillance) combined with other therapy; (3) reviews, comments or conference abstracts; (4) publications not in English; and (5) animal experiments.

### Data Collection and Assessment of the Risk of Bias in the Included Studies

A formalized table was independently used to extract the data from each paper by YW and BM. The following information was included: (1) authors; (2) publication year; (3) study design; (4) setting (single center/multicenter); (5) number of patients; (6) patient sex; (7) patient age; (8) length of BE; (9) multifocal dysplasia; (10) rate of technical success (en bloc); (11) rate of LGD progression to HGD and/or EAC; (12) rate of CE-D and CE-IM; and (13) adverse events.

Cochrane analysis was conducted to assess the risk of bias for the RCTs (10). Five aspects of bias—selection bias, performance bias, detection bias, attrition bias and reporting bias—were evaluated. The results of the assessment of the included RCTs are provided in **Supplementary Table 3**.

### Primary and Secondary Outcomes

The primary outcome of this study was the rate of progression of BE-LGD to HGD and/or EAC in patients treated with RFA or endoscopic surveillance. The secondary outcome was the rate of CE-D and CE-IM in patients treated with RFA and endoscopic surveillance. Adverse events were also extracted and evaluated.

### Statistical Analysis

RevMan 5.3 (Cochrane) was used for the statistical analysis. The Mantel–Haenszel random effects model and risk ratios (RRs) were used. The random-effects model was used for all outcomes, because it provided more conservative estimates and was tailored to multicenter studies in which heterogeneity is typically present (11). I^2^ was used to evaluate heterogeneity, and I^2^ > 50% and P < 0.05 were considered thresholds for significant heterogeneity. Subgroup analyses were performed for progression of BE-LGD to HGD and BE-LGD to EAC. If the number of included studies exceeded ten, assessment of publication bias was planned to be performed. All statistical values are reported with 95% confidence intervals (CI). Moreover, the overall assessment of certainty of evidence was conducted according to the Grading of Recommendations Assessment, Development and Evaluation approach (GRADE) (12) by using GRADEprofiler (version 3.6).

### IRB Approval

This meta-analysis is not related with any patient privacy or related information, so there is no need for IRB approval.

## Results

### Search Results

**Figure 1:**
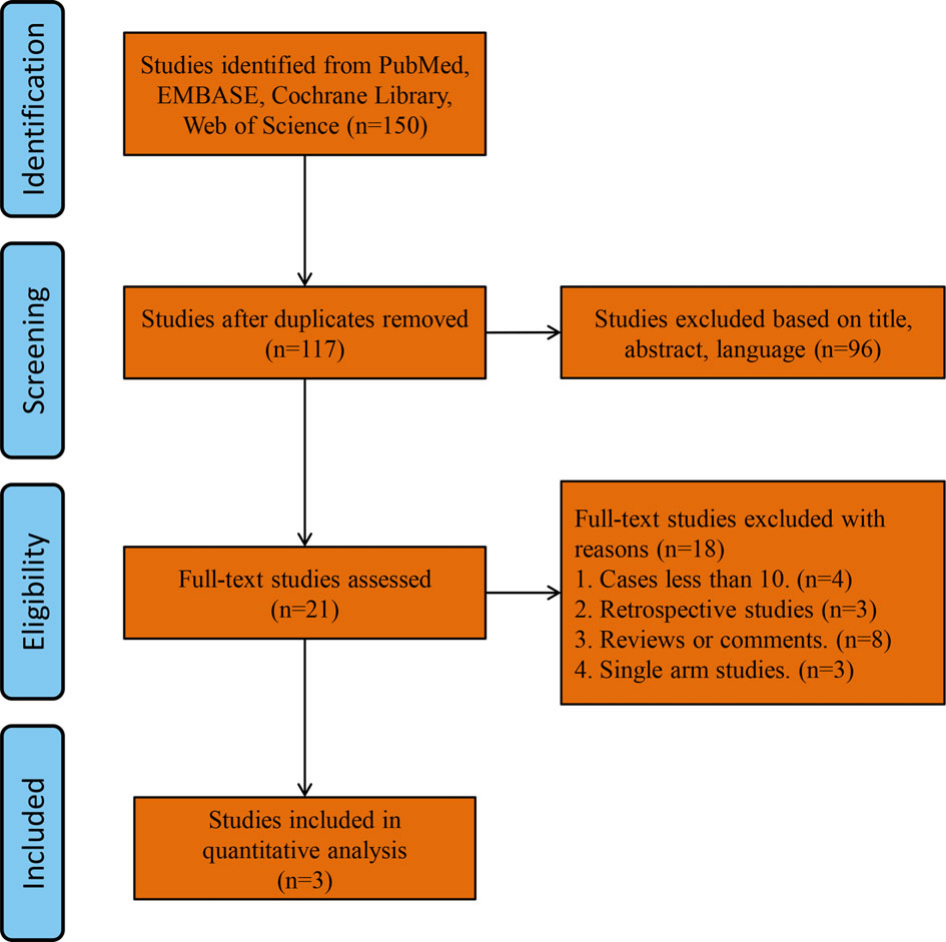
Flow chart of this meta-analysis.

A total of 150 studies were found by searching the PubMed, Cochrane Library and Web of Science databases. The study flowchart is shown in **Figure 1**. Thirty-three duplicate studies were excluded, and an additional 96 studies were removed for reasons associated with the title, abstract and language. Twenty-one records were eligible for full text review. Further screening was conducted on the basis of the inclusion and exclusion criteria, and the reasons for exclusion are shown in **Figure 1**. Finally, only three RCTs were eligible for the final meta-analysis (1, 2, 13). The main characteristics of the included studies are shown in **Table 1**, and the summary characteristics of the patients in the included studies are shown in **Table 2**.

**Table 1:** The main characteristics of the included studies.

Authors	Year	Country	Setting	Study design	Patients (n)		Mean age (year)		Male/Female	
					RFA^*^	Surveillance	RFA	Surveillance	RFA	Surveillance
1.Shaheen	2009	USA	Multicenter	RCT	42	22	66.3 ± 1.4	64.6 ± 1.9	33/9	19/3
2.Phoa	2014	Netherlands	Multicenter	RCT	68	68	63 ± 10	63 ± 9	55/13	61/7
3.Barret	2021	France	Multicenter	RCT	40	42	62.8 ± 10.2	61.8 ± 9.9	36/4	10/2

*RFA, Radiofrequency ablation.

**Table 2:** The summary characteristics of the patients in the included studies.

Authors	Length of Barrett’s esophagus (cm)		Multifocal dysplasia		Time since diagnosis of Barrett’s esophagus (year)		Time since diagnosis of dyplasia (year)	
	RFA	Surveillance	RFA	Surveillance	RFA	Surveillance	RFA	Surveillance
1.Shaheen	4.6 ± 0.4	4.6 ± 0.5	32	13	5.8 ± 0.7	5.2 ± 1.0	2.2 ± 0.5	2.4 ± 0.6
2.Phoa	median 4 (2-8)	median 4 (3-6)	NA	NA	median 5 (2-10)	median 7 (3-11)	median 1 (0-5)	median 2 (0-5)
3.Barret	NA	NA	NA	NA	6.1 ± 5.6	5.5 ± 5.0	2.2 ± 3.2	2.2 ± 2.4

NA, not available.

### Methodological Quality of Included Studies (Risk of Bias)

Methodological quality was evaluated for the included studies, as shown in **Supplementary Table 3**. The concealment of randomization and allocation were clearly described in all included studies. The RFA device was the same across studies (HALO^360^ and HALO^90^, using the BarrX system from Medtronic). The methods of endoscopic biopsy of specimens were nearly the same across studies, with collection every 1 or 2 cm and from any visible abnormalities. All biopsies were assessed by two expert pathologists; if the readings were discordant, a third pathologist was assigned to review the results. Hence, the detection bias among the studies was also low. All studies reported the reasons for loss to follow-up and further treatment in detail, thus making the attrition bias and reporting bias low. Hence, the overall risk of bias in the included studies was low.

### Primary Outcome

**Figure 2:**
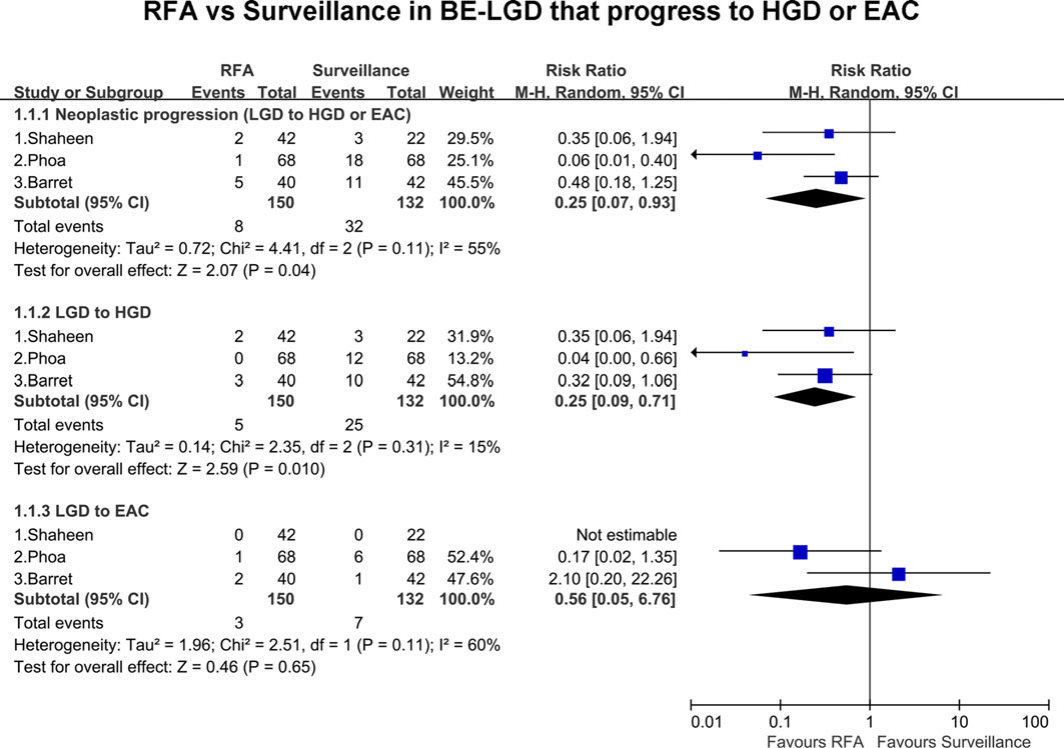
Pooled risk of BE-LGD progression to HGD or EAC (RFA vs. surveillance).

All included studies, including 282 patients, presented the rate of progression of BE-LGD to HGD and/or EAC. Overall, eight patients progressed to HGD and EAC in the RFA group compared with 32 in the endoscopic surveillance group. The pooled estimate of the rate of neoplastic progression of BE-LGD to HGD or EAC was much lower in the RFA group than the endoscopic surveillance group (RR, 0.25; 95% CI, 0.07–0.93; P = 0.04), with moderate heterogeneity (I^2^ = 55%); however, the heterogeneity was not statistically significant (P = 0.11) (**Figure 2**). Subgroup analysis of the final progression grade was performed. The pooled rate of progression of BE-LGD to HGD was much lower in the RFA group than the endoscopic surveillance group (RR, 0.25; 95% CI, 0.07–0.71; P = 0.01), with low heterogeneity (I^2^ = 15%) (**Figure 2**). The pooled rate of progression of BE-LGD to EAC was slightly lower in the RFA group than the endoscopic surveillance group (RR, 0.56; 95% CI, 0.05–6.76), and the result was not statistically significant (P = 0.65) (**Figure 2**). GRADE indicated low certainty of evidence, owing to the large confidence intervals, and publication bias was not assessed because of the small number of studies (**Supplementary Table 4**).

### Secondary Outcomes

**Figure 3:**
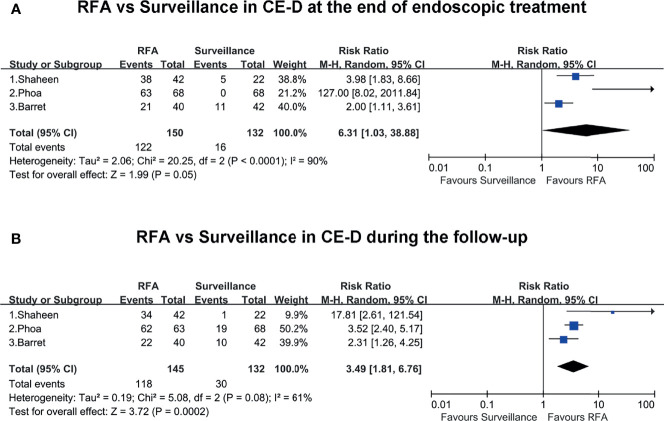
**(A)** RFA vs. surveillance for CE-D at the end of endoscopic treatment. **(B)** RFA vs. surveillance for CE-D during the follow-up.

All the included studies reported the rates of CE-D in the RFA group and surveillance group at the end of endoscopic treatment and during the follow-up. At the end of endoscopic treatment, the rate of CE-D was higher in the RFA group than the endoscopic surveillance group (RR, 6.31; 95% CI, 1.03–38.88; P = 0.05), and the result was not statistically significant (**Figure 3A**). However, during follow-up, the rate of CE-D was higher in the RFA group than the endoscopic surveillance group (RR, 3.49; 95% CI, 1.81–6.76; P < 0.01), with moderate heterogeneity (I^2^ = 61%) (**Figure 3B**). The certainty of evidence according to GRADE was low and moderate for CE-D at the end of endoscopic treatment and during follow-up, respectively (**Supplementary Table 4**).

**Figure 4:**
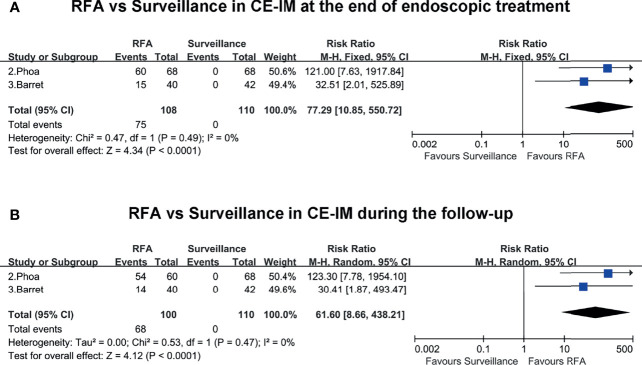
**(A)** RFA vs. surveillance for CE-IM at the end of endoscopic treatment. **(B)** RFA vs. surveillance for CE-IM during the follow-up.

Two included studies presented the rates of CE-IM in the RFA group and surveillance group at the end of endoscopic treatment and during the follow-up. At the end of endoscopic treatment, the rate of CE-IM was much higher in the RFA group than the endoscopic surveillance group (RR, 77.29; 95% CI, 10.85–550.72; P < 0.01), with low heterogeneity (I^2^ = 0%) (**Figure 4A**). Similarly, during the follow-up, the rate of CE-IM was also clearly higher in the RFA group than the endoscopic surveillance group (RR, 61.6; 95% CI, 8.66–438.21; P < 0.01), with low heterogeneity (I^2^ = 0%) (**Figure 4B**). The certainty of evidence according to GRADE was moderate both for CE-IM at the end of endoscopic treatment and during the follow-up (**Supplementary Table 4**).

### Adverse Events

All three included studies presented the adverse events, which could be classified into mild adverse events, severe adverse events and esophageal strictures (**Table 3**). In general, the adverse events occurred more frequently in the RFA group than the surveillance group. Chest pain was the most common adverse event, and was mild and treated by analgesics. Fever, bleeding, vomiting and nausea were the most common severe adverse events; however, the number of patients with these symptoms was small, and the symptoms could be treated conservatively. Esophageal stricture requires treatment with an endoscopic procedure. The mean/median sessions of endoscopic dilation ranged from 1 to 2.6 (**Table 3**). No severe adverse events and esophageal strictures were reported in the endoscopic surveillance group. Moreover, no perforations or procedure-related deaths occurred.

**Table 3:** The information of adverse event.

Authors	Adverse events					
	Mild adverse event		Severe adverse event		Esophageal stricture	
	RFA	Surveillance	RFA	Surveillance	RFA	Surveillance
1.Shaheen	40 patients have chest pain.	20 patients have chest pain	3^*^ (upper gastrointestinal hemorrhage, chest-pain 8 days after RFA, chest discomfort and nausea immediately after RFA	0	5 patients required dilation (mean 2.6 dilation)	0
2.Phoa	3 patients have small mucosal laceration; One has retrosternal pain 3 weeks after RFA.	0	3 (abdominal pain 4 days after RFA; one patient has two adverse event: bleeding 7 days after RFA followed by endoscopic resection, later the same patients was dilated for stricture and developed fever and chills)	0	8 patients required dilation (median 1 dilation (IQR^#^, 1-2)	0
3.Barret	9 patients have chest pain	0	3 patients have vomiting; 4 patients have fever.	0	1 patient (no information for treatment)	0

*This number of adverse event contained all the patient treated with RFA.

IQR, interquartile range.

## Discussion

In the past 30 years, the incidence of esophageal adenocarcinoma has clearly increased (14), and Barrett’s esophagus is the main cause. Because of the different grades of progression of BE, the optimal treatment varies and is a matter of controversy. For nondysplastic intestinal metaplasia, endoscopic surveillance is feasible and rational. Endoscopic resection of visible lesions and ablation of residual BE are widely accepted treatments for BE-HGD and early EAC (5, 15). However, substantial evidence is lacking regarding the optimal intervention for BE-LGD, whose histopathological diagnosis is challenging and whose course is uncertain. To our knowledge, only three RCTs have compared RFA and endoscopic surveillance in patients with a confirmed diagnosis of BE-LGD. Hence, this meta-analysis aimed to compare the efficacy and safety between these two procedures.

Several retrospective studies have also reported results of interest (1, 7); however, the unavoidable bias in the study design made them unsuitable for inclusion in our analysis. All three included RCTs had high study design quality, from calculation of the sample size to administration of randomization, and reported the details of results. The pooled results indicated that RFA, compared with endoscopic surveillance, indeed decreased the risk of BE-LGD progression to BE-HGD or EAC by a total of up to 75%. Subgroup analysis revealed that RFA reduced the risk of progression of BE-LGD to BE-HGD by 75%. However, the reduced risk of progression of BE-LGD to EAC was not statistically significant, possibly because the progression of BE-LGD occurs in a stepwise manner. More time might have been needed to observe the natural progression of BE-HGD to EAC, and thus only a small number of patients showed BE-LGD progression to EAC. In the endoscopic surveillance group, only seven patients progressed to EAC, whereas, 25 patients progressed to BE-HGD. Although the pooled estimate of RFA in reducing the risk of LGD to HGD is encouraging, the results should be interpreted with caution. The certainty of evidence according to GRADE is low, owing to the large confidence interval and unavailable assessment of publication bias. Phoa et al. (2) reported that 28% of the control group in their study presented no dysplasia progression, findings similar to those of Shaheen et al., in which 26% of the control remained in LGD without showing dysplasia during the follow-up. Consequently, the same proportion of patients might theoretically exist in the RFA group with low risk of dysplasia progression, and might have been overtreated by RFA. Furthermore, Barret et al. (13) reported a 31% rate of spontaneous clearance of LGD in their included patients. Together, these results illustrate the difficulty in diagnosing BE-LGD and making appropriate treatment decisions. In fact, Phoa et al. (2) analyzed the predictors of progression in the endoscopic surveillance group and found that the number of years after BE diagnosis, the number of endoscopies with dysplasia before inclusion and the length of circumferential BE were independent predictors. However, the study did not find predictors of the spontaneous regression of BE. Therefore, as suggested by Krishnamoorthi (16), development of a risk assessment tool including the clinical risk factors and biomarkers is urgently needed to enable appropriate decisions for each patient.

Both the pooled estimates of CE-D and CE-IM at the end of endoscopic treatment and during the follow-up were higher in the RFA group than the endoscopic surveillance group. Of note, the rate of CE-D clearly decreased from the end of endoscopic treatment to the follow-up (RR: from 6.31 to 3.49). A potential explanation is that 14 more patients had spontaneous clearance of dysplasia in the surveillance group during the follow-up compared with at the endoscopic treatment. Barret et al. (13) described a spontaneous clearance rate (31%) slightly higher than those reported by Shaheen et al. (1) and Phoa et al. (2) (22.7% and 27.9%, respectively), whereas much higher rates of degeneration ranging from 34% to 75% have been reported in previous studies (6, 17, 18). To exclude sampling error, all three studies conducted repeated strict biopsy measurements; moreover, at least two expert pathologists evaluated the results. All these findings illustrate that BE-LGD should be prudently chosen for indications in patients under surveillance, given the uncertain course of disease, progression.

RFA is considered a safe and efficacious technique for BE-LGD with non-visible lesions, as compared with endoscopic resection (19, 20). The most common adverse event after the RFA procedure is chest pain, which can be treated conservatively. Sporadic severe adverse events, such as bleeding, fever, nausea and vomiting, may occur. Esophageal stricture is another adverse event almost always requiring endoscopic dilation.

This is the first meta-analysis of the efficacy and safety of RFA vs. endoscopic surveillance for BE-LGD based on RCTs. This study has several strengths. First, the quality of all included studies was high; moreover, the study design was consistent among the included RCTs, thus decreasing the heterogeneity to the greatest extent possible. Second, all studies clarified the power analysis used to calculate the sample size, thereby supporting the validity of the results. Third, the GRADE evaluation system was used to estimate the certainty of the evidence, beyond the statistical results. However, some study limitations must also be noted. First, the number of included studies was small, with a small number of cases overall. Second, the heterogeneity of some pooled results was moderate to substantial, although there is no statistically significant of these heterogeneity, prudent interpretation for these results. Finally, because all the studies were conducted in expert medical centers, they appear to be difficult to generalize to community-practice settings.

In conclusion, RFA appears to decrease the risk of progression of BE-LGD to BE-HGD, while simultaneously achieving a higher rate of CE-D and CE-IM, both at the end of endoscopic treatment and during follow-up. However, given the uncertain course of LGD and the possibility of esophageal stricture after RFA, the treatments should be fully considered and weighed. More studies and more cases are needed to elucidate the ability of RFA to decrease the risk of progression of BE-LGD to higher grades.

## Data Availability Statement

The original contributions presented in the study are included in the article/**Supplementary Material**. Further inquiries can be directed to the corresponding author.

## Author Contributions

YW and BM designed the study idea and the study methodology. SY and WL conducted the research and analyzed data. PL participated in the coordination of the study and provided specific support in quantitative data analysis. YW and PL wrote the manuscript. All authors read and approved the version of the manuscript.

## Funding

YW has received research funding by 345 Talent Project from Shengjing Hospital of China Medical University.

## Conflict of Interest

The authors declare that the research was conducted in the absence of any commercial or financial relationships that could be construed as a potential conflict of interest.

## Publisher’s Note

All claims expressed in this article are solely those of the authors and do not necessarily represent those of their affiliated organizations, or those of the publisher, the editors and the reviewers. Any product that may be evaluated in this article, or claim that may be made by its manufacturer, is not guaranteed or endorsed by the publisher.
