# Supplementary material for: Efficacy and Safety of Radiofrequency Ablation vs. Endoscopic Surveillance for Barrett’s Esophagus With Low-Grade Dysplasia: Meta-Analysis of Randomized Controlled Trials
Source: Front Oncol. 2022 Feb 28;12:801940. doi: 10.3389/fonc.2022.801940 (PMC8920305; doi:10.3389/fonc.2022.801940)
Supplement: Supplementary file 1 [file Table_1.docx]

**Supplementary table 1 Literature search strategy in different database**

| **Database** | **Search strategy** | **No. of literature** |
| --- | --- | --- |
| **PubMed** | ("barrett esophagus"[MeSH Terms] OR "barrett esophagus"[All Fields] OR "barrett's esophagus"[All Fields] OR "barrett oesophagus"[All Fields] OR "barrett metaplasia"[All Fields]) AND ("radiofrequency ablation"[MeSH Terms] OR ("radiofrequency"[All Fields] AND "ablation"[All Fields]) OR "radiofrequency ablation"[All Fields] OR "RFA"[All Fields]) AND (("barrett esophagus"[MeSH Terms] OR "barrett esophagus"[All Fields] OR "barrett's esophagus"[All Fields] OR "barrett oesophagus"[All Fields] OR "barrett metaplasia"[All Fields]) AND ("radiofrequency ablation"[MeSH Terms] OR ("radiofrequency"[All Fields] AND "ablation"[All Fields]) OR "radiofrequency ablation"[All Fields] OR "RFA"[All Fields])) AND ("RCT"[All Fields] OR "Randomized Controlled Trial"[All Fields]) | 36 |
| **Web of Science** | "barrett esophagus" OR "barrett's esophagus" OR "barrett oesophagus" OR "barrett metaplasia" (Topic) and "radiofrequency ablation" OR "RFA" (Topic) and "RCT" OR "Randomized Controlled Trial" (Topic) | 49 |
| **Cochrance** | (("barrett esophagus" OR "barrett's esophagus" OR "barrett oesophagus" OR "barrett metaplasia" )):ti,ab,kw AND (("radiofrequency ablation" OR "RFA")):ti,ab,kw AND (("RCT" OR "Randomized Controlled Trial")):ti,ab,kw | 65 |
