# Supplementary material for: Efficacy and Safety of Radiofrequency Ablation vs. Endoscopic Surveillance for Barrett’s Esophagus With Low-Grade Dysplasia: Meta-Analysis of Randomized Controlled Trials
Source: Front Oncol. 2022 Feb 28;12:801940. doi: 10.3389/fonc.2022.801940 (PMC8920305; doi:10.3389/fonc.2022.801940)
Supplement: Supplementary file 3 [file Table_3.docx]

**Supplementary Table 3 Risk of bias assessment of RCTs using the Cochrane Collaboration tool**

| **Study** | **Random sequence generation** | **Allocation concealment** | **Performance bias** | **Detection bias** | **Attrition bias** | **Reporting bias** |
| --- | --- | --- | --- | --- | --- | --- |
| Shaheen, 2009 | Low | Low | Low | Low | Low | Low |
| Phoa, 2014 | Low | Low | Low | Low | Low | Low |
| Barret, 2021 | Low | Low | Low | Low | Low | Low |

RCT, Randomized controlled trial
