# Supplementary material for: Efficacy and Safety of Radiofrequency Ablation vs. Endoscopic Surveillance for Barrett’s Esophagus With Low-Grade Dysplasia: Meta-Analysis of Randomized Controlled Trials
Source: Front Oncol. 2022 Feb 28;12:801940. doi: 10.3389/fonc.2022.801940 (PMC8920305; doi:10.3389/fonc.2022.801940)
Supplement: Supplementary file 4 [file Table_4.docx]

**Supplemenatry table 4 Certainty of evidence based on GRADE**

| **Quality assessment** | | | | | | | **No of patients** | | **Effect** | | **Quality** | **Importance** |
| --- | --- | --- | --- | --- | --- | --- | --- | --- | --- | --- | --- | --- |
|  |  |  |  |  |  |  |  |  |  |  |  |  |
| **No of studies** | **Design** | **Risk of bias** | **Inconsistency** | **Indirectness** | **Imprecision** | **Other considerations** |  |  | **Relative (95% CI)** | **Absolute** |  |  |
| **BE-LGD to HGD or EAC** | | | | | | | | | | | | |
| 3 | randomised trials | no serious risk of bias | no serious inconsistency | no serious indirectness | serious^1^ | reporting bias^2^ | 8/150  (5.3%) | 32/132  (24.2%) | RR 0.25 (0.07 to 0.93) | 182 fewer per 1000 (from 17 fewer to 225 fewer) | ⊕⊕OO LOW | CRITICAL |
|  |  |  |  |  |  |  |  | 0% |  | - |  |  |
| **RFA vs Surveillance in CE-D at the end of endoscopic treatment** | | | | | | | | | | | | |
| 3 | randomised trials | no serious risk of bias | no serious inconsistency | no serious indirectness | serious^1^ | reporting bias^2^ | 122/150  (81.3%) | 16/132  (12.1%) | RR 6.31 (1.03 to 38.88) | 644 more per 1000 (from 4 more to 1000 more) | ⊕⊕OO LOW | CRITICAL |
|  |  |  |  |  |  |  |  | 0% |  | - |  |  |
| **RFA vs Surveillance in CE-D during the follow-up** | | | | | | | | | | | | |
| 3 | randomised trials | no serious risk of bias | no serious inconsistency | no serious indirectness | no serious imprecision | reporting bias^2^ | 118/145  (81.4%) | 30/132  (22.7%) | RR 3.49 (1.81 to 6.76) | 566 more per 1000 (from 184 more to 1000 more) | ⊕⊕⊕O MODERATE | CRITICAL |
|  |  |  |  |  |  |  |  | 0% |  | - |  |  |
| **RFA vs Surveillance in CE-IM at the end of endoscopic treatment** | | | | | | | | | | | | |
| 2 | randomised trials | no serious risk of bias | no serious inconsistency | no serious indirectness | no serious imprecision | reporting bias^2^ | 75/108  (69.4%) | 0/110  (0%) | RR 77.29 (10.85 to 550.72) | - | ⊕⊕⊕O MODERATE | CRITICAL |
|  |  |  |  |  |  |  |  | 0% |  | - |  |  |
| **RFA vs Surveillance in CE-IM during the follow-up** | | | | | | | | | | | | |
| 2 | randomised trials | no serious risk of bias | no serious inconsistency | no serious indirectness | no serious imprecision | reporting bias^2^ | 68/100  (68%) | 0/110  (0%) | RR 61.6 (8.66 to 438.21) | - | ⊕⊕⊕O MODERATE | CRITICAL |
|  |  |  |  |  |  |  |  | 0% |  | - |  |  |

^1^ Due to confidence interval is large.
^2^ Publication bias not assessed due to small number of studies.
